# Unlocking New Treatment Possibilities for Metastatic Endometrial Cancer With KRAS G12C Mutation

**DOI:** 10.1155/crom/7966703

**Published:** 2025-11-28

**Authors:** Bana Antonios, Oyepeju Abioye, Seon Jo Park, Gene Finley

**Affiliations:** ^1^Allegheny Health Network-Allegheny General, Pittsburgh, Pennsylvania, USA; ^2^Allegheny General Hospital, Pittsburgh, Pennsylvania, USA

**Keywords:** adagrasib, endometrial cancer, KRAS G12C, targeted therapy

## Abstract

Endometrial carcinoma (EC) is a heterogeneous malignancy with diverse molecular subtypes that influence prognosis and treatment response. While conventional therapies such as surgery, chemotherapy, and radiation remain the mainstay of treatment, recurrent and metastatic EC poses significant therapeutic challenges, particularly in aggressive histologic subtypes like clear cell carcinoma. Advances in genomic profiling have revealed that KRAS mutations occur in approximately 10%–30% of EC cases, with the KRAS G12C variant representing a rare but potentially targetable alteration. KRAS G12C inhibitors, including sotorasib and adagrasib, have revolutionized the treatment landscape for certain malignancies, particularly non–small cell lung cancer (NSCLC) and colorectal cancer (CRC), where they have received FDA approval. The efficacy of these agents in other KRAS G12C–mutated solid tumors remains under investigation, with limited clinical data available in endometrial cancer. To date, only three documented cases have reported responses to KRAS G12C inhibitors in EC, highlighting the need for further exploration of targeted strategies in this setting. Here, we present a unique case of a 77-year-old woman with metastatic endometrial clear cell carcinoma who exhibited a durable response to adagrasib after progressing on multiple lines of standard treatment. This case highlights the potential clinical utility of KRAS G12C inhibitors in EC and highlights the importance of molecular profiling in identifying actionable mutations that may guide treatment decisions. This report, contributing to the limited body of evidence that includes three prior cases evaluating the role of sotorasib and adagrasib across several solid malignancies, highlights the clinical and translational relevance of adagrasib in advancing precision-targeted therapy for KRAS G12C–mutated tumors.

## 1. Introduction

KRAS-G12C mutations are increasingly recognized as actionable targets in various solid tumors, with adagrasib and sotorasib emerging as key therapies. These targeted agents have received FDA approval for treating KRAS G12C–mutated non–small cell lung cancer (NSCLC) and colorectal cancer (CRC), with sotorasib approved in May 2021 and adagrasib following in December 2022 [1, 2]. Their success has spurred clinical interest in evaluating their efficacy across other KRAS G12C–mutated solid tumors. However, despite the expanding role of KRAS G12C inhibitors in these malignancies, their relevance in gynecologic cancers remains less defined. While KRAS mutations are found in approximately 10%–30% of endometrial carcinomas (ECs), the specifically targetable G12C variant is less common. To date, only three documented cases have reported successful treatment outcomes with these agents in KRAS G12C–mutated endometrial cancer—two with adagrasib and one with sotorasib [3].

## 2. Case Presentation

We present the case of a 77-year-old woman with metastatic endometrial clear cell carcinoma. She was initially diagnosed following a total hysterectomy with bilateral salpingo-oophorectomy and pelvic lymph node dissection, with pathology confirming FIGO Stage IIIC disease. After surgery, she completed six cycles of adjuvant chemotherapy with carboplatin and paclitaxel, along with concurrent pelvic radiation.

Despite initial effective treatment, she eventually developed metastatic disease. She subsequently received multiple lines of therapy per standard guidelines, including platinum-based chemotherapy (carboplatin and paclitaxel), anti-VEGF antibodies (bevacizumab), tyrosine kinase inhibitors (lenvatinib) combined with anti-PD-1 immunotherapy (pembrolizumab), a pegylated doxorubicin-based regimen, and a topoisomerase inhibitor (topotecan).

After four cycles of a topoisomerase inhibitor, a computed tomography (CT) scan demonstrated disease progression with peritoneal carcinomatosis, mediastinal and retroperitoneal lymphadenopathy, and multiple hepatic lesions. Concurrently, her CA-125 level was markedly elevated, reaching 3149 U/mL. Despite these findings, she remained largely asymptomatic, with an Eastern Cooperative Oncology Group (ECOG) performance status of 1–2 at treatment initiation and throughout her course.

Molecular profiling of her surgical specimen at diagnosis, performed using a next-generation sequencing (NGS), identified a PIK3CA mutation with a variable allelic frequency (VAF) of 25.8% and a KRAS G12C mutation with a VAF of 72.7% and microsatellite stable (MSS).

**Figure 1:**
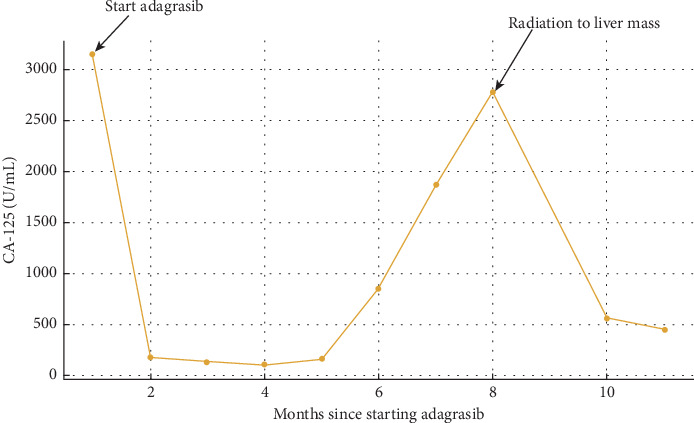
CA-125 levels over time during adagrasib therapy.

Considering her favorable molecular profile, the high KRAS G12C mutation burden, and the absence of other approved systemic treatment options, we initiated adagrasib therapy. She initially was unable to tolerate the standard dose of 600 mg twice a day due to Grade 2 nausea and vomiting, requiring dose reduction to 200 mg twice a day which she was able to tolerate well and was eventually increased to 400 mg twice a day. Three months after starting treatment, her restaging CT scan showed a partial response in all metastatic sites, including mediastinal lymph nodes, multiple hepatic lesions, and peritoneal carcinomatosis. This radiologic response correlated with her CA-125 trend, which dropped to a nadir of 108 U/mL as demonstrated in Figure 1.

Approximately 6 months into adagrasib treatment, her tumor markers began to rise, suggesting potential disease progression. A restaging PET scan revealed overall stable disease, with the exception of notable progression in one large liver mass. A biopsy confirmed that the histology was consistent with her primary malignancy and reflected by CA-125 increase to 2777 U/mL. Given her overall stable disease, we decided to treat the single site of progression with local radiotherapy. Meanwhile, she continued to tolerate adagrasib well, with no significant side effects. After completing radiation, her CA-125 dropped to 307 U/mL while she was still on adagrasib at the time of this report, marking 11 months of therapy.

## 3. Discussion

EC is a highly aggressive malignancy, often resistant to conventional treatment and associated with a poor prognosis. While our patient initially responded well to first-line therapy, her disease eventually progressed through multiple lines of standard treatments [4].

This resistance is likely driven by the tumor's molecular profile, which frequently includes mutations in TP53, ARID1A, PIK3CA, and PTEN pathways, making EC difficult to treat and emphasizing the need for better therapies. Given its high risk of recurrence and metastasis, EC is often managed aggressively. Standard treatment typically involves surgery, including hysterectomy with bilateral salpingo-oophorectomy, lymph node dissection, and pelvic washings for staging and tumor debulking. For high-risk patients, adjuvant therapy is commonly recommended, with platinum-based chemotherapy with or without radiotherapy forming the cornerstone of systemic treatments [5, 6].

Discovered in 1982, the KRAS mutations are known drivers of oncogenesis in a range of malignancies. The KRAS protein activates various signaling pathways, including the MAPK and PI3K/AKT pathways. When mutated, this results in uncontrolled cellular proliferation, leading to increased cellular growth and resistance to apoptosis [1, 6]. A retrospective study of 7870 patients with endometrial cancer identified KRAS mutations in 16% of cases, with G12D and G12V being the most common subtypes. However, the G12C variant, which is found in 10%–13% of NSCLCs, 3%–4% of CRCs, and 1%–2% of biliary and pancreatic cancer, was detected in 88 patients, representing approximately 1.1% of the entire study population [7].

Historically, KRAS mutations were considered nonactionable due to the lack of suitable binding sites for therapeutic targeting [8]. However, in 2021, sotorasib became the first KRAS G12C inhibitor to receive accelerated approval following a Phase II trial in patients with advanced NSCLC who had previously undergone at least one line of therapy. A year and a half later, adagrasib, another selective KRAS G12C inhibitor, was approved for the same patient population based on data from a single-arm study [9, 10].

Adagrasib binds irreversibly to the KRAS G12C mutant by forming a covalent bond with the cysteine residue at Codon 12, locking KRAS in its inactive GDP-bound state. Resistance can arise through multiple mechanisms, including secondary KRAS mutations (e.g., G12D, G13D, Q61H, and Y96C) and KRASG12C allele amplification. Bypass pathway activation has also been observed via alterations in MET, NRAS, BRAF, MAP 2K1, RET, and oncogenic fusions involving ALK, RAF1, and FGFR3. Additionally, histologic transformation and loss-of-function mutations in NF1 or PTEN may further contribute to therapeutic resistance [11].

In 2024, both sotorasib and adagrasib received FDA approval for the treatment of KRAS G12C–mutated metastatic CRC in combination with the anti-EGFR monoclonal antibodies panitumumab and cetuximab, respectively. Beyond NSCLC and CRC, these inhibitors have also demonstrated promise in other malignancies [2, 12].

Pharmacokinetic analyses show that adagrasib possesses a longer half-life and greater oral bioavailability and superior central nervous system (CNS) penetration when compared to sotorasib, which may enhance its activity in those with brain metastases. The two agents also differ in resistance dynamics: Sotorasib is associated with earlier emergence of secondary KRAS mutations, while adagrasib has demonstrated more durable responses, including in previously treated patients [13].

Hong et al. explored the use of sotorasib in solid malignancies before its official approval for NSCLC. Among 129 patients studied, the majority had NSCLC or CRC. Of 28 patients in the study with other tumor types, four achieved a partial response, including one patient with endometrial cancer who maintained a response for 6.9 months. Meanwhile, four patients experienced disease progression, while the remaining patients had stable disease [14].

Later, Bekaii-Saab et al. evaluated the efficacy of adagrasib in 57 patients with KRAS G12C–mutated tumors, excluding those with NSCLC and CRC. Nearly half of the patients achieved stable disease, while an objective partial response was observed in 35.1% of cases, including two out of three patients with endometrial cancer. The slightly higher number of patients with endometrial cancer in this study influenced our decision to choose adagrasib for our patient rather than sotorasib. The median progression-free survival (PFS) for the overall study population was 7.4 months, while patients with endometrial cancer had a median PFS of 5.6 months [3].

This study was particularly noteworthy as it demonstrated a partial response in seven out of 21 patients with pancreatic ductal adenocarcinoma and five out of 12 patients with biliary tract cancer, both of which are known to be challenging cancers to treat. This highlights the potential of targeted therapies across various cancer types and paves the way for future treatment options.

This study, along with our reported case, highlights the significant role of NGS in various solid tumors. By identifying actionable mutations such as KRAS G12C, NGS directly guided personalized therapy decisions for our patient, enabling the selection of a targeted therapy that would have otherwise been overlooked in standard treatment algorithms. This underscores the value of molecular profiling in precision oncology and sets the stage for future clinical trials [8].

## 4. Conclusion

This case contributes to the growing evidence supporting the potential role of targeted therapy with available KRAS G12C inhibitors in the treatment of EC. With limited therapeutic options for patients with advanced disease who have progressed on standard treatments, molecular profiling to identify such actionable mutations is becoming increasingly critical. While early reports show promising outcomes, larger studies are necessary to define the efficacy and role of KRAS G12C inhibitors in this setting. However, resistance mechanisms remain a significant challenge, indicating the need for continued research into combination strategies to enhance treatment outcomes, particularly for patients with refractory disease.

## Data Availability

The data that support the findings of this study are available on request from the corresponding author. The data are not publicly available due to privacy or ethical restrictions.
